# A New Industry—Commercializing a Social Waste-Product

**Published:** 1910-04

**Authors:** 


A NEW INDUSTRY—COMMERCIALIZING A SOCIAL WASTE-PRODUCT.—Wherever in our complex social, political, commercial, industrial or religious life there is a "want" there are always men and means ready to supply that want. Advertising has become a fine art. Our magazines are richly and handsomely illuminated with pretty pictures of every commodity needed, from a collar button to a fifty thousand dollar auto. But there is a "crying" want long overlooked, a "want" of tremendous possibilities as to profit, to which our attention has just been directed.

We have received a beautifully gotten up album, issued by a maternity sanitarium out West, in which are offered a large and select assortment of "superior babies for adoption" [*sic:* title page]. Samples are shown by photo-engravings (after the manner of Easter hats), of the several, or rather, numerous spring styles of babies, properly designated, "Heart's Fondest Hope," "Answer to Prayer," "Nurse's Pride," "A Dear Little Dimpled Darling," "Papa's Right Bower" ("Papa" anonymous), "A Ray of Sunshine," etc., all pretty little babies that appeal irresistibly to the heart of a babyless woman. This maternity sanitarium advertises that it "has an exceptionally high grade of children on hand. Practically all of them are of unfortunate parentage" (that is, of ruined girls). The album advertises that there are 2,000,000 childless homes in the United States. These 2,000,000 homes the sanitarium proposes to supply with babies at so much per, and can give purchasers a very select list of paternities to choose from. Get the best. The goods advertised are crosses between working girls, as a rule, the unprotected "lambs," and social wolves in sheeps' clothing; among which (the sanitarium names with pride) are: "Ministers, Sunday school teachers, physicians, wholesale merchants, lawyers, bankers," etc. "A complete list," says the advertisement, "would include practically all the better avocations, both commercial and professional." These, of course, come higher. If one (babyless family) would be content with a cheaper and less aristocratic article, the sanitarium offers a very select lot of miscellaneous paternity, a kind of bargain counter as it were of "business college teachers, music teachers, concert players, clerks, drummers, compositors (printers), real estate men, etc. [I quote from the advertisement.] And—this demand for babies to supply 2,000,000 homes shall be met, even if the "ministers, Sunday school teachers, physicians and lawyers" have to work overtime. Something is said about charges, but it is not payment for the baby, of course, but just enough to cover cost. A release guarantee and (confidentially the name of the pater) go with each baby. These cost money. Among the many subheads in the album the most interesting are: "How to Get a Baby" (correspondence schools will catch on, and, first thing you know, they will be teaching it by mail). "Method of Adoption." "Where Duty Lies," etc. Seriously, the plan is a good one and to be commended. We wish it every success. The output of social waifs is unusually large, it is intimated, and doubtless will increase under the teachings of the free-love literature called novels that the United States postal laws permit to go through the mails: "Three Weeks," for instance, and "Marriage *a la mode*," and "Trial Marriages," etc.

---

THE SEMI-ANNUAL MEETING of the Fifth District (Bexar) Medical Society, March 22 ult., was made notable by the presence of the famous Jno. B. Murphy, who read a paper on "Joint Surgery From a Practitioner's Standpoint," and R. H. L. Bibb, of Mexico, an old Texan and general favorite with the Texas doctors. Dr.

Bibb is authority on leprosy. His paper, "Early Bone Lesion in Leprosy," was listened to with profound attention and interest, especially seeing that the intelligent and earnest efforts of the Texas medical profession to "do something" with the lepers in Texas and their success in getting a bill through the last Legislature providing for an asylum where the fifty or more cases now at large, a menace to the people, could be segregated, isolated, were negatived by our wise Governor who could not be induced to carry out the provisions of the bill even after it had become a law. Dr. Bibb declared that "leprosy is contagious, infectious and communicable under conditions not yet understood, and that by isolation and segregation, and by that only, can the disease be eradicated from the list of human ills; that is the lesson taught by the experience of the world."

Dr. M. J. Bleim, of San Antonio, read a paper on "The Growing Disuse of Alcohol in Therapeutics." He declared that there has been a rapid diminution in the use of stimulants in medical practice, and produced statistics in support of his contention; that the medical profession has almost universally discarded alcohol as a therapeutic agency.

There were other notable papers read—amongst them one by Dr. Theo. Y. Hull, of San Antonio, on the "Treatment of Tuberculosis"; one by Prof. Keiller, of the Texas University Medical College, on the "Anatomy of the Rectum" in relation to operations for cancer of that viscus.

The session closed with a smoker and vaudeville performance on the roof of the St. Anthony Hotel. Dr. A. R. Bowman, of Uvalde, the President of the Association, was toast-master.

---

Senator Owen, of Oklahoma, appears before the United States Senate as champion of the cause of a Department of Public Health with a Secretary in the Cabinet, as against the proposal to keep the great public health interest as a mere bureau in one of the Departments. He has introduced a bill in the Senate exactly in accordance with the views, wishes and advice of the united medical profession as voiced by the American Medical Association, the American Public Health League, the Committee of One Hundred, etc., and has backed it up by a powerful and unanswerable argument. A copy of this great speech can be had for the asking. All honor to the brave and talented Senator.

It would seem to require no argument to convince any thoughtful person of the truth of our contention, so fittingly expressed by the great Roosevelt—"the public health is the nation's most valuable asset,"—and yet—it must occupy a secondary position in a department of secondary importance in comparison. We come next to pigs and cabbages in the opinion of our wise lawmakers.

